# Open chromatin dynamics reveals stage-specific transcriptional networks in hiPSC-based neurodevelopmental model

**DOI:** 10.1016/j.scr.2018.03.014

**Published:** 2018-03-31

**Authors:** Siwei Zhang, Winton Moy, Hanwen Zhang, Catherine Leites, Heather McGowan, Jianxin Shi, Alan R. Sanders, Zhiping P. Pang, Pablo V. Gejman, Jubao Duan

**Affiliations:** a Center for Psychiatric Genetics, NorthShore University HealthSystem, Evanston, IL 60201, USA; b Department of Psychiatry and Behavioral Neuroscience, University of Chicago, IL 60637, USA; c Department of Neuroscience and Cell Biology and Child Health Institute of New Jersey, Rutgers University, New Brunswick, NJ 08901, USA; d Biostatistics Branch, Division of Cancer Epidemiology and Genetics, National Cancer Institute, Bethesda, MD 20892, USA

## Abstract

Chromatin accessibility to transcription factors (TFs) strongly influences gene transcription and cell differentiation. However, a mechanistic understanding of the transcriptional control during the neuronal differentiation of human induced pluripotent stem cells (hiPSCs), a promising cellular model for mental disorders, remains elusive. Here, we carried out additional analyses on our recently published open chromatin regions (OCRs) profiling at different stages of hiPSC neuronal differentiation. We found that the dynamic changes of OCR during neuronal differentiation highlighted cell stage-specific gene networks, and the chromatin accessibility at the core promoter region of a gene correlates with the corresponding transcript abundance. Within the cell stage-specific OCRs, we identified the binding of cell stage-specific TFs and observed a lag of a neuronal TF binding behind the mRNA expression of the corresponding TF. Interestingly, binding footprints of NEUROD1 and NEUROG2, both of which induce high efficient conversion of hiPSCs to glutamatergic neurons, were among those most enriched in the relatively mature neurons. Furthermore, TF network analysis showed that both NEUROD1 and NEUROG2 were present in the same core TF network specific to more mature neurons, suggesting a pivotal mechanism of epigenetic control of neuronal differentiation and maturation. Our study provides novel insights into the epigenetic control of glutamatergic neurogenesis in the context of TF networks, which may be instrumental to improving hiPSC modeling of neuropsychiatric disorders.

## 1. Introduction

Human induced pluripotent stem cells (hiPSCs)-differentiated neurons have served as a promising model to gain insight into the molecular and cellular mechanisms of genetic risk related to mental disorders (Panchision, 2016; Wen et al., 2016). Comparing to human brains and the emerging brain organoids (Pasca et al., 2015; Rigamonti et al., 2016; Birey et al., 2017; Qian et al., 2016; Quadrato et al., 2017), hiPSC-derived monolayer neurons are relatively homogeneous and, therefore, advantageous in assaying developmental stage-specific and cell type-specific phenotypic changes, as well as the underlying molecular signatures (Wen et al., 2016; Brennand et al., 2011). By using different combinations of growth factors and small molecules in culture media, hiPSCs can be efficiently differentiated into specific types of neurons, including midbrain dopaminergic (Kriks et al., 2011), cortical glutamatergic (Shi et al., 2012a), and GABAergic inhibitory interneurons (Liu et al., 2013; Maroof et al., 2013; Nicholas et al., 2013), as well as into microglia (Muffat et al., 2016). As an alternative to media supplemented with growth factors, forced expression of exogenous transcription factors (TFs) has also been applied to quickly differentiate hiPSCs into functional neuronal lineages, such as the rapid differentiation of excitatory neurons via forced expression of NEUROD1 or NEUROG2 (Vierbuchen et al., 2010; Zhang et al., 2013) or the GABAergic inhibitory interneurons via forced expression of ASCL1 and DLX2 (Yang et al., 2017). These methods often give rise to neurons with variable homogeneity and functional maturity. Hence, a mechanistic understanding of the temporal epigenetic control of neuronal differentiation from hiPSCs would greatly facilitate the optimisation of hiPSC models.

Multiple aspects are known to determine the fate and trajectory of neuronal differentiation (Birling and Price, 1995; Patterson and Nawa, 1993), and transcriptional regulation has long been considered to play a pivotal role in the process. Transcription is strongly influenced by the accessibility of TFs to chromatin (Degner et al., 2012; Thurman et al., 2012). In turn, cellular differentiation is a process of epigenetic transition of chromatin states from multipotent stem cells to differentiated cells (Chen and Dent, 2014), accompanied by the changing accessibility of Open Chromatin Regions (OCRs) to TF occupancy. TFs are essential for neuronal differentiation. However, while it is well known that chromatin remodeling (Ronan et al., 2013) influences neurogenesis and neural differentiation, the relationship between chromatin state dynamics and neural development, especially in hiPSC-derived neurons, remains poorly understood.

**Fig. 1:**
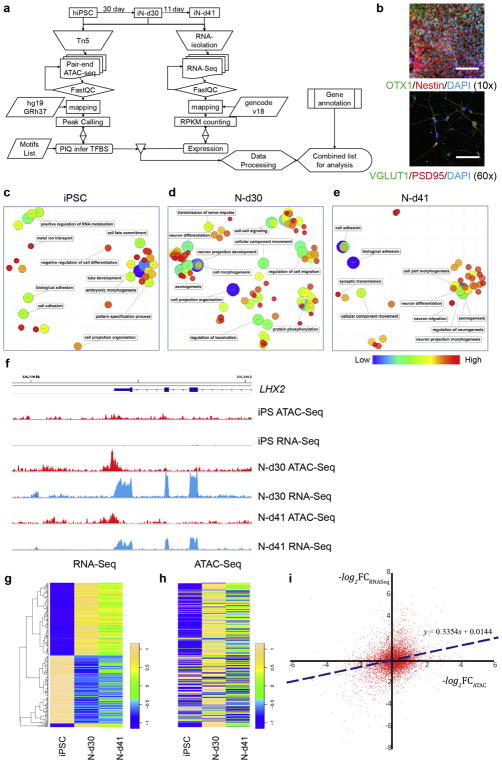
Open chromatin dynamics and correlation with gene expression during cortical neuron differentiation. (A) Flowchart showing the cell preparation, ATAC-seq open chromatin mapping, and RNA-seq analysis. (B) Immunofluorescence (IF) staining of cortical NSCs (OTX1+/Nestin+; top panel) and N-d41 glutamatergic neurons (VGLUT1+/PSD95+; bottom panel). Scale bar = 120 μm and 20 μm, respectively. (C–E) GO-term enrichment analysis of cell stage-specific OCR peaks during cortical neuronal differentiation from hiPSCs, showing the GO-term (biological process) enrichment for open chromatin peaks at different specific stages (C) hiPSC, (D) N-d30, (E) N-d41. The GO-term enrichment was generated by DAVID (v6.7). The enriched GO-terms at FDR < 0.05 were clustered and visualized by REVIGO. Scale bar indicates the statistical significance of the enrichment (blue = lowest *P* values, i.e. highest significance of enrichment). (F) ATAC-seq peaks (upper, red) and RNA-seq peaks (lower, blue) near the 5′-end of LHX2, a forebrain-specific gene in hiPSCs, N-d30, and N-d41 neurons, showing the strong correlation between ATAC-seq and RNA-seq peak intensities. (G–H) Heat maps showing the expression ratio (normalized RPKM) of the 272 genes (G) and their normalized ATAC-seq read counts ratio at its promoter-TSS peaks (H). The 272 genes are most variable ones with >4-fold of expression changes between hiPSC and N-d30 stages (FDR < 0.005); (I) XY scatter plot showing the correlation between ATAC-seq read-count fold-change within Promoter-TSS and the expression fold-change (RNA-seq). The negative logarithmic values the r ATAC-seq read-count fold-change within Promoter-TSS and the expression fold-change (RNA-seq) between hiPSC and N-d41 stages were plotted.

With the cortical glutamatergic neurons efficiently derived from hiPSCs (Shi et al., 2012a; Shi et al., 2012b), we have recently performed a global mapping of OCRs using the Assay for Transposase-Accessible Chromatin with high throughput sequencing (ATAC-seq) (Buenrostro et al., 2013) and identified abundant cell stage-specific OCRs (Forrest et al., 2017). Here, with the previously mapped OCR profiles (Forrest et al., 2017) and newly analyzed RNA-seq from the same experiment (Forrest et al., 2017), we examined the correlations of the dynamic changes of OCRs with cell stage-specific gene pathways and transcriptomics changes in hiPSC-derived neurons at various stages of differentiation process. We further assembled the neuronal stage-specific TF networks through a genome-wide inference of TF-binding footprints in OCRs (Fig. 1A). We found that the accessibility of the predicted TF Binding Sites (TFBSs) is highly dynamic during hiPSC-derived neuronal differentiation, and such dynamic changes are crucial for the TF network regulation and cell lineage determination.

## 2. Materials and methods

An Extended Experimental Procedures has been provided as part of the Supplementary Materials and Methods for details.

### 2.1. hiPSC lines, cell culture, and glutamatergic neuronal differentiation

We used the hiPSC line derived from GM01835 for open chromatin mapping. The study has been approved by the NorthShore University HealthSystem IRB. mTeSR1 media (StemCell) were used to culture hiPSCs in Geltrex-coated Petri dishes (ThermoFisher). Glutamatergic neuronal induction and differentiation were performed according to (Shi et al., 2012b) with minor modifications to make compatible with feeder-free culture environment. Dorsomorphin and SB431532 were added for neural induction. Cells were collected at their respective stages (hiPSC, N-d30, and N-d41) for ATAC-seq (Forrest et al., 2017) and RNA-seq. Specifically, we have two replicates per stage (an average of day-27 and day-33 neurons was considered as a hypothetical day-30 neuronal stage due to high correlation of day-27 and day-33 ATAC-seq data) (Forrest et al., 2017) for the ATAC-seq and RNA-seq data, with the exception that the RNA-seq data of N-d41 included only one replicate.

### 2.2. Next-generation sequencing and data analysis

For ATAC-seq, cells were collected on the designated culture day. Cell nuclei were immediately isolated and subjected to transposon reaction (Buenrostro et al., 2013). Processed DNA was stored at −20 °C before the assembly of sequencing libraries. Poole2d libraries were sequenced on a HiSeq 2500 with 2 × 50 bp paired-end setting at the University of Minnesota Genomics Center (UMGC). The detailed statistics of the ATAC-seq have been documented in our previous study (Forrest et al., 2017). Briefly, we used 2 × 50 bp pair-end (PE) sequencing to obtain ATAC-seq data at 30 M reads per replicate, 2 replicates per stage, and 6 samples in total. The collected raw PE reads range from 22 M bp to 25 M bp per sample. Subsequently, Hotspot analysis was performed on each individual replicate. HOMER and PIQ were used to estimate the enrichment of TF motifs within OCRs or TFBFs in each cell stage, respectively (Heinz et al., 2010; Sherwood et al., 2014). TFBF enrichment was further evaluated using Fisher’s exact test. CytoScape was used to construct TF network using PIQ-inferred TFBF data. For RNA-seq, MirVana kit (ThermoFisher) was used to extract total RNAs. RNA-seq was performed at the UMGC on an Illumina HiSeq 2500 using v4 chemistry to obtain single-end 50 bp reads, generating approximately 30 M reads per sample. The reads were mapped to the human exome (GENCODE v18), and gene-level expression was calculated as RPKM.

### 2.3. Constructing TF regulatory networks

TF networks specific to N-d30 and N-d41 were assembled by using CytoScape. The master nodes are N-d30 and N-d41 specific TFs that have footprints inferred by PIQ with a cut-off score of 0.9 and those that form the most connected TF network.

## 3. Results

### 3.1. Dynamic changes of open chromatin during hiPSC differentiation into glutamatergic neurons inform cell stage-specific gene networks

Changes in chromatin openness during cell differentiation affect transcriptional activity and gene network activity. We have previously obtained ATAC-seq and RNA-seq data in differentiating glutamatergic neurons at three different stages: hiPSC stage, 30 days (NSC and early-stage neurons; an average of day 27 and day 33 neurons) and 41 days (relatively mature neurons) post neuronal induction (N-d30 and N-d41, Fig. 1A) (Forrest et al., 2017). The glutamatergic neuronal differentiation method (Shi et al., 2012a; Shi et al., 2014) generated relatively homogenous NSC and excitatory neurons (N-d41) as assayed by immunofluorescence staining (Fig. 1B and Fig. S1) and by single-cell gene expression analysis as shown in our original study (Forrest et al., 2017). By inspecting the open chromatin peaks called by Hotspot, we identified abundant cell stage-specific OCRs (Forrest et al., 2017). The number of peaks generated by Hotspot varies between different cell stages (Forrest et al., 2017): 27,685 in hiPSC, 57,413 in N-d30, and 31,836 in N-d41 stage. Of these, 8861 peaks are hiPSC-specific, 26,012 peaks are N-d30-specific, and 5006 peaks are N-d41-specific. Here, to test whether the cell stage-specific OCRs were correlated with gene networks specific to different cell fates, we performed Gene Ontology (GO) enrichment analysis of genes nearest to those stage-specific OCRs. By clustering the enriched GO-terms with FDR < 0.05 (Fig. 1C–E and Fig. S2) (Gene Ontology, 2015; Ashburner et al., 2000), we observed a distinctive switch of the gene regulation program from hiPSCs to neurons. Indeed, the hiPSC-specific peaks demonstrated the characteristic traits of pluripotency and self-renewal, with enriched GO terms such as embryonic morphogenesis, negative regulation of cell differentiation, and cell fate commitment (Fig. 1C). In contrast, neuron-specific peak groups showed characteristics of differentiated neuronal identity, with enrichment of GO terms such as neuron differentiation, axonogenesis, and cellular component movement (Fig. 1D–E). Moreover, close examination showed that N-d41 represents a more mature stage of neuronal differentiation than at N-d30, as noted by the enrichment of GO terms such as synaptic transmission and neuron projection morphogenesis. With GREAT (McLean et al., 2010), a tool for genomic interval-based enrichment analysis, we observed similar enrichment of GO-terms related to more mature neurons in N-d41, e.g., axon guidance and axonogenesis (Table S3).

To further quantify the OCR openness of these cell stage-specific peaks, we compared the sequencing reads within each ATAC-seq peak, which have been normalized against the total read count of each sample, across hiPSC, N-d30, and N-d41 stages by using EdgeR (Robinson et al., 2010). An FDR of 5% was used as a cut-off to determine the significance of the differences in chromatin accessibility of OCRs between stages. For those OCRs that showed significant changes between stages, based on their directional changes during the hiPSC → N-d30 and N-d30 → N-d41 transitions (up/down), we subsequently divided the peaks into six independent groups and performed GO term analysis for each group (Fig. S2A–F and Table S1). Consistent with the stage-specific enrichment results, each of the five plotted groups demonstrated a distinctive identity, whilst the down-down group excluded due to its very small number of peaks (Fig. S2a). The down-flat group, which contained peaks whose openness was reduced during the hiPSC → N-d30 transition but remained unchanged during the N-d30 → N-d41 transition, represented a collection of cell growth and development-related gene sets. The flat-down group showed enriched GO-terms related to cellular components movement and cell growth, consistent with the substantially reduced cell growth and movement at a later stage (N-d41) of neuronal differentiation. The up-flat and flat-up groups were both characterized by their pro-neuronal identity. The last group, up-up, also possessed pro-neural and pro-neuronal identity as noted by the enrichment of neuron differentiation and axonogenesis. Together, these results suggest that neuronal differentiation from hiPSCs is accompanied by temporal OCR dynamics of different sets of cell stage-specific genes that would subsequently determine the neuronal fate.

### 3.2. Open chromatin state dynamics is correlated with transcriptomic changes during neuronal differentiation

OCRs often overlap with cis-regulatory sequences, and thus may directly influence gene transcription and cell differentiation (Fullard et al., 2017). Hence, we hypothesised that OCR openness and gene expression are correlated with neuronal differentiation from hiPSCs. Considering the complexity of open chromatin-mediated gene regulation, e.g. multiple OCRs for the same gene, we focused on the OCRs flanking the core promoter-TSS (transcriptional starting site). We first visually compared the quantile-normalized RNA-seq data (in Reads Per Kilobase per Million mapped reads, RPKM) against the normalized ATAC-seq read counts of promoter-TSS OCRs for some specific genes at individual genomic loci and in general, we observed a concordant directional changes of OCR peak (as revealed by ATAC-seq) and gene expression pattern (as revealed by RNA-seq). For example, LHX2, a forebrain-specific gene (Porter et al., 1997; Zhang et al., 2014) that was specifically expressed in differentiated neurons, demonstrated a robust ATAC-seq peak at core promoter-TSS in N-d30 and N-d41 neurons, but not in hiPSCs in which its expression and ATAC-seq peak were both minimal (Fig. 1F). For a set of 272 genes that showed most variable expression (>4-fold expression differences between hiPSCs and N-d30; FDR < 0.005 by EdgeR), we further examined their concordant changes of expression and chromatin accessibility in all three cell stages by plotting their hierarchical clustering and heat map of expression levels (Fig. 1G) and OCR ATAC-seq counts flanking the core promoter-TSS (Fig. 1H). Although a concordant pattern can be visually identified between RNA-seq and ATAC-seq data, the correlation for each cell stage seemed to be moderate (Fig. 1G and H). This observation was supported by the moderate Pearson’s correlation of their expression level and the promoter-TSS OCR openness at each cell stage (*R* = 0.21 and *P =* 2.2 × 10^−3^ for hiPSCs, *R* = 0.42 and *P* = 2.1 × 10^−10^ for N-d30, *R* = 0.41 and *P* = 6.0 × 10^−10^ for N-d41) (Fig. S3).

We further examine the genome-wide correlation of the dynamic changes of OCR and gene expression by comparing the fold-changes (FCs) of mRNA expression and promoter-TSS ATAC-seq reads between hiPSCs and N-d41 stages. We found a positive moderate correlation between the two (*R* = 0.28, *P* < 2.2 × 10^−16^) (Fig. 1I). To further statistically confirm the positive correlation, we selected all genes with FC > 2 in expression levels or OCR openness (Fig. 1I), and tested whether the genes with same directional changes are significantly more than those with opposite directional changes by using Fisher’s exact test. This test confirmed the positive correlation between the dynamic changes of mRNA expression and promoter-TSS OCR openness (Fisher’s exact test, 2-sides, *P* < 2.2 × 10^−16^) (Fig. 1I). Our observed moderate correlation of promoter ATAC-seq peak intensity with gene expression level in hiPSCs and the differentiated neurons is not unexpected and is consistent with the results of a previous study conducted in mouse embryos (Wu et al., 2016), suggesting a conserved mechanism for epigenetic control of gene expression in early neurodevelopmental stages.

To further gain biological insights on genes that showed concordant dynamic changes between OCR openness and gene expression during hiPSC neuronal differentiation, we further performed GO-term enrichment analysis. For the same groups of genes defined by their ATAC-seq peak dynamics (up/down) (Table S1), only the up-flat and down-flat groups gave sufficient number of genes that also showed the same expression dynamic changes for such enrichment analysis (Table S4). Genes in the group sharing the same up-flat dynamics between ATAC-seq and RNA-seq were found highly enriched for GO-terms related to neuron differentiation and neurogenesis (e.g., axon guidance and axonogenesis) (FDR < 0.05), and with higher folds of enrichments than those in the same group of ATAC-seq peaks (4–8 folds vs. 2–3 folds) (Table S5). In contrast, although with a larger number of genes showing the same down-flat dynamics between ATAC-seq peaks and RNA-seq, this group of genes did not show any enrichment of GO-term (FDR < 0.05) (Table S6), with only nominally significant GO-terms related to non-neuronal developmental processes, metabolic process and cell signalling (Table S6). These results suggest although a large number of non-neuronal developmental genes may have chromatin open in hiPSCs and underwent reduction of chromatin accessibility during hiPSC neuronal differentiation (i.e., down-flat) (Table S6), most of which did not show concordant expression reduction due to very low or no expression in hiPSC (Table S4). On the contrary, most neurodevelopmental genes show concordant dynamic changes between gene expression and chromatin accessibility during hiPSC → neuron differentiation (upregulated).

### 3.3. Dynamic changes of OCRs are correlated with cell stage-specific TF binding events

**Fig. 2:**
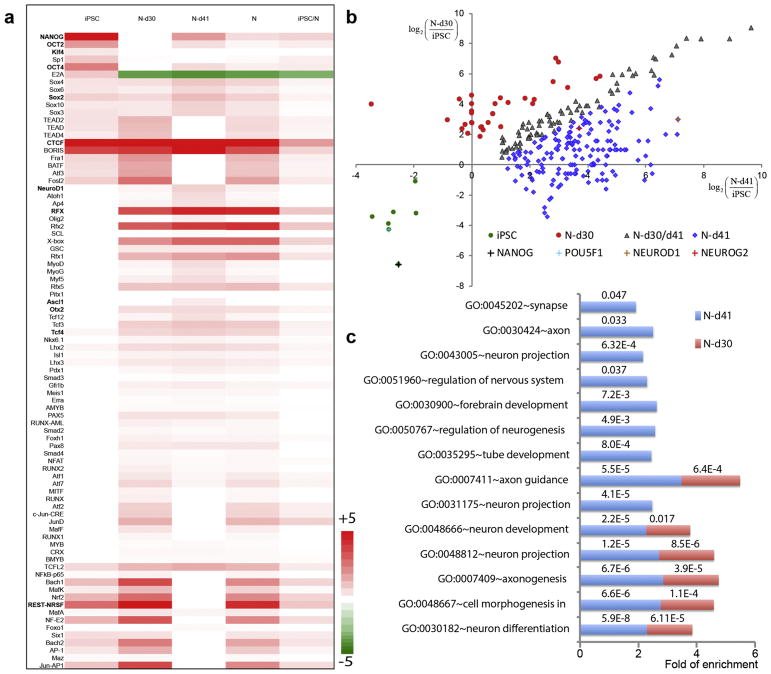
Cell-type specific TFBSs enriched in cell-type specific open chromatin peaks. (A) 264 curated TF motifs generated by HOMER with a significance of enrichment at *P* value < 10^−20^ (Bonferroni corrected) in hiPSC, N-d30, or N-d41 specific peaks are plotted, and the color indicates the scale of enrichment (red) or depletion (green). White = no significant enrichment. N: hiPSC-derived neurons (N-d30 and N-d41 combined). (B) TFBS specific to hiPSC (green), N-d30 (red), N-d41 (blue), or shared in N-d30/N-d41 (gray) as defined by >2-fold difference of number of TFBFs between cell types (Fisher’s exact test *P* < .05; Bonferroni corrected). Highlighted are several most enriched TFs and previously known cell-type specific TFs in each cell stage. (C) Neuronal GO terms enriched (FDR < 0.05; DAVID v6.7) in gene targets of N-d41 and N-d30 cell type-specific TFs. More enriched neuronal GO terms that are representative of more mature neurons (e.g. synapse, axon, and tube development) and higher fold of enrichment are found for TF targets in N-d41 than in N-d30.

TF binding events at OCRs are crucial to cell differentiation (Degner et al., 2012; Thurman et al., 2012). Whilst some TFs (e.g. NEUROD1, NEUROG2, ASCL1) have been shown to be essential for glutamatergic neuronal differentiation (Vierbuchen et al., 2010; Zhang et al., 2013), the absence of a genome-wide view of the key TFs and their regulatory networks has hampered the understanding of the epigenetic control of this differentiation process. Global open chromatin profiling enables an unbiased analysis of cell stage-specific TF binding events occurring at OCRs. We first used HOMER, a tool for sequence-based motif enrichment analysis (Heinz et al., 2010), to identify the enriched TF motifs at cell-stage specific OCR sites. We found OCRs in each cell stage have different sets of TF motifs enriched (Fig. 2A). hiPSC-specific OCRs had the least number of enriched TFs, which were characterized by pluripotency maintenance (e.g. NANOG). Notably, the TF with binding motif most enriched at N-d41 was found with NEUROD1 (*P* = 2.4 × 10^−46^, Fisher’s exact test), a TF that was known for its ability to rapidly induce the differentiation of excitatory neuron from stem cells. In addition, motifs of other TFs (e.g. ASCL1, LHX2) known to act together with NEUROD1 to convert fibroblasts into neurons, were also enriched in neuron-specific OCRs (Fig. 2A) (Vierbuchen et al., 2010).

Since the HOMER-based TF motif enrichment analysis did not reflect the actual TF occupancy at OCR, we then used both motif- and open chromatin peak pattern-based Protein Interaction Quantification (PIQ) tool (Sherwood et al., 2014) to predict TFs that physically occupied OCRs. We inferred TF-binding “footprints” (TFBF) from our ATAC-seq data, yielding 2.1 M, 2.9 M, and 2.2 M of TFBFs for hiPSCs, N-d30, and, N-d41 stages(Forrest et al., 2017). Out of the 1357 TFs that were inferred to have footprints, 300 TFs were either specific to one of the three cell stages only, hiPSC (*n* = 7), N-d30 (*n* = 33), or N-d41 (*n* = 185), or shared by N-d30 and N-d41 (*n* = 75), with a cut-off threshold of more than two-fold difference in the numbers of TFBFs between cell types (Bonferroni corrected *P* < .05) (Fig. 2B and Table S2). In addition, a null hypothesis that the stage-specific TF distrubtion pattern can be attributed to random sampling was rejected by Fisher’s exact test (*P* = 8.93 × 10^−7^). Consistent with the enrichment of TF motif analysis by HOMER (Fig. 2A), TFBFs of NANOG and NEUROD1 were most enriched in hiPSCs and N-d41, respectively. TFBFs of the TF TEAD1 and its cognate DNA-binding partner YAP are known to promote the expansion of the neural progenitors (Cao et al., 2008), and were most enriched in N-d30 (Fig. 2B). Moreover, TFBFs of NEUROG2, another TF that can induce rapid differentiation of excitatory neurons (Vierbuchen et al., 2010; Zhang et al., 2013), were also highly enriched in N-d41 (Fig. 2B). Importantly, using the gene targets of N-d30 and N-d41 cell-specific TFs for GO term analysis (Huang da et al., 2009), we found enriched neuronal GO terms representing mature neurons, such as synapse, axon, and tube development, as well as a higher fold of enrichment in the N-d41 than the N-d30 stage, suggesting a continuous differentiation and maturation from the N-d30 phase to the N-d41 phase, the latter reflecting a more mature stage of differentiated neurons (Fig. 2C).

Interestingly, while TFs enriched at the N-d41 stage exhibited a characteristic neuron-specific expression pattern, the mRNA expression level of N-d41 stage-specific TFs peaked at the N-d30 stage (7/7; binominal *P* < .05, Fig. S4B–C), suggesting the actual binding events of these TFs were lagged behind their peak expression. Similarly, TFs most enriched at N-d30 neurons often showed a detectable level of expression in hiPSCs (Fig. S4), although their peak expression was not at hiPSC stage. While the observed lag of actual TF binding events was not unexpected, (i.e. a TF has to be expressed before it plays a functional role), such temporal asynchronisation of TF binding events and their mRNA expression may be related to the epigenetic regulation at the open chromatin level during neuronal differentiation. The lag of the peak TF occupancy of most neuron-specific TFs compared to their transcriptional peaks suggests that it is the timing of TF occupancy in open chromatin, but not the timing of TF expression, that serves as the hallmark of a developmental stage.

### 3.4. TF network analyses imply a pivotal role for NEUROD1 and NEUROG2 in glutamatergic neuronal differentiation

**Fig. 3:**
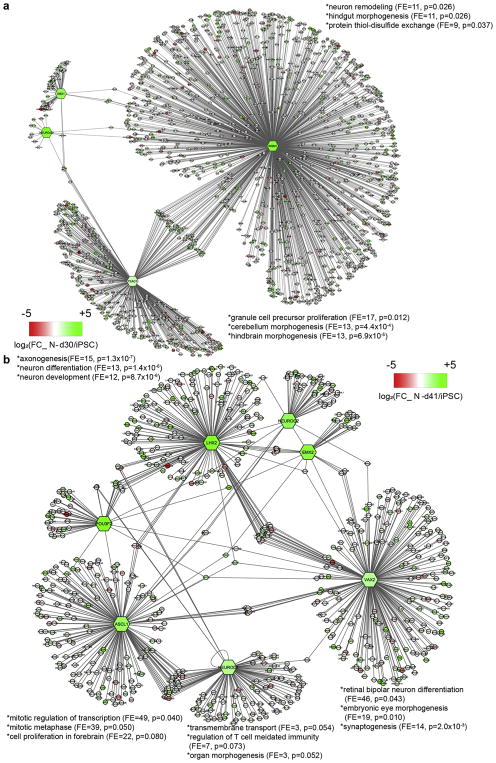
TF transcriptional networks that consist of PIQ-inferred TFs and their occupied target genes in N-d30 and N-d41. (A) Most connected TF network in N-d30. The top three most enriched (FE = fold enrichment) GO-terms with a *P* value < .05 (analyzed by DAVID v6.7)) were listed for a TF network (if available). The scale bar shows the expression fold-change (FC; N-d30/hiPSC). (B) Most connected TF network in N-d41. The networks were generated in CytoScape using the PIQ-inferred TFBS data with a cut-off score of 0.9, and the gene expression FC data from RNA-seq. Hexagon: node regulators; circles: regulated genes.

Having inferred TFBFs, we next identified key TF networks functional during glutamatergic neuronal differentiation. To make a more biologically meaningful inference, we focused on those N-d30 and N-d41 stage-specific TFs that also showed high levels of neuronal expression (see Methods). The most connected N-d30 TF network included TEAD1, INSM1, and DBX1 as master nodes (Fig. 3A), of which TEAD1 was the most enriched TF in N-d30 (Fig. 2B) and has been known to promote the expansion of neural progenitors (Cao et al., 2008). Among the N-d30-specific TFs, INSM1 had the most binding targets (*n* = 1340) in the N-d30 network and had been known to promote neurogenesis and regulate the proliferation-differentiation balance in developing brain (Lorenzen et al., 2015; Monaghan et al., 2017). Interestingly, we found that NEUROG2, a TF that can induce rapid neuronal differentiation (Zhang et al., 2013), was a target of INSM1 and also shared common target gene with another master node TEAD1 in the N-d30 network (Fig. 3A), supporting an important role of NEUROG2 in early stages of neuronal differentiation.

The most connected N-d41 TF network contained both NEUROD1 and NEUROG2, of which NEUROD1 shared the most targets (*n* = 24) with another N-d41-specific TF, ASCL1 (Fig. 3B). Together with EMX2, POU3F2, LHX2, and VAX2, they constitute the master TF nodes in the N-d41 TF network. Each subgroup of this TF network reflects a unique aspect of neuronal differentiation. For example, LHX2 and VAX2 targets are highly enriched in neuronal GO terms, while NEUROD1 and ASCL1 targets are more related to cell cycle and mitosis. In addition, many NEUROD1 targets, such as TPBG, GPC3, and FAT4, are also well-known for their roles during neuronal differentiation and maturation (Hu et al., 2015; Pilia et al., 1996; Cappello et al., 2013). Moreover, NEUROG2 connects to multiple master TF nodes including ASCL1, EMX2, LHX2, and POU3F2, either directly or through shared targets (Fig. 3B). Therefore, both TF networks (N-d30 and N-d41) support a pivotal role of NEUROD1 and NEUROG2 during the differentiation of glutamatergic neurons.

**Fig. 4:**
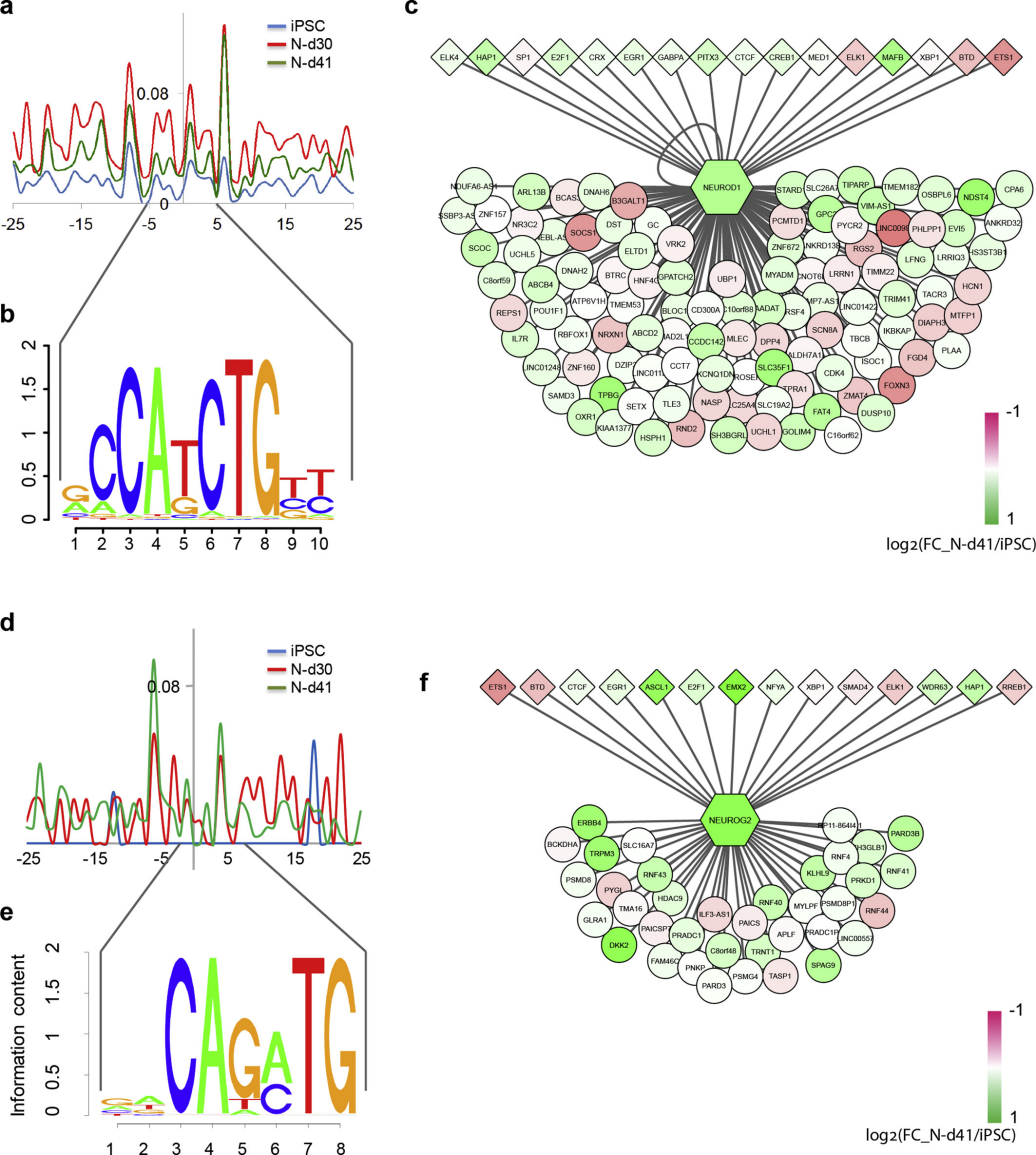
ATAC-seq tag intensity around PIQ-inferred TFBFs and the extracted regulatory network of NEUROD1 and NEUROG2. (A) ATAC-seq tag intensity plot at the PIQ-inferred TFBFs of NEUROD1 in each cell stage. Note that the N-d41 tag intensity plot (green) showed a distinctive “dip” that is characteristic of NEUROD1 binding footprint comparing to N-d30 (red). hiPSC tag intensity plot (blue) appears to be background noise. (B) Visualized 10-bp NEUROD1 binding motif sequence. (C) The NEUROD1 regulatory network including the TFs that regulate (i.e. bind to) NEUROD1 (diamonds) and the gene targets of NEUROD1 (circles). Scale bar shows the expression fold changes (FC; N-d41/hiPSC). (D) ATAC-seq tag intensity plot at the PIQ-inferred binding footprints of NEUROG2 in each cell stage. Note the significantly increased tag density at N-d41 stage (green) comparing to the N-d30 stage (red), also the nearly flat tag density at hiPSC stage (blue). (E) Visualized 8-bp NEUROG2 binding motif sequence. (F) The NEUROG2 regulatory network including the TFs that regulate NEUROG2 (diamonds) and the gene targets of NEUROG2 (circles). All the networks were generated in CytoScape using the PIQ-inferred TFBS data and the gene expression FC data.

Given the central role of NEUROD1 and NEUROG2 in the N-d41 TF network, we further verified the empirical neuron-specific occupancy at their corresponding binding motifs (Fig. 4A–B and D–E) and identified the upstream TFs directly binding to their regulatory genomic regions (Fig. 4C and F). By directly comparing the ATAC-seq tag density at regions flanking the genome-wide TF-binding motifs of NEUROD1 and NEUROG2 in each cell stage, we found that N-d41 showed the most characteristic binding patterns (i.e. a typical dip between the two ATAC-peaks) of NEUROD1 (Fig. 4A–B) and NEUROG2 (Fig. 4D–E), while hiPSCs did not show any specific TF-binding with N-d30 binding signals in between. This phenomenon is consistent with their dynamic changes of DNA occupancy across different cell stages and their central roles in the N-d41 TF network. However, it should be mentioned that NEUROD1 and NEUROG2 share a degree of similarity within their binding motifs, noticeably at the 3rd–4th and 7th–8th base (Fig. 4B, E). To identify the TFs that may directly regulate NEUROD1 and NEUROG2, we examined the PIQ-inferred TFBFs within 100 kb upstream of the TSSs of NEUROD1 and NEUROG2. We found both TFs are regulated by several other TFs known to be crucial for neurogenesis (Fig. 4C and F). Interestingly, over half of the upstream regulatory TFs of NEUROD1 and NEUROG2 overlap (BTD, CTCF, E2F1, EGR1, ELK1, ETS1, HAP1, and XBP1), suggesting a shared regulatory program that determines excitatory neuronal fate. Among those overlapping regulatory TFs of NEUROD1 and NEUROG2, HAP1 is known to be important in postnatal neurogenesis (Xiang et al., 2014; Xiang et al., 2015), while ETS1 and ELK1 have partially redundant activities in the *Ciona* anterior neural plate of gastrulating embryos (Gainous et al., 2015). Moreover, we found that NEUROD1 targets a putative promoter adjacent to the TSS (−213 bp of NM_002500) of NEUROD1 itself, which provides a mechanism at the open chromatin level for a previously reported autoregulation of NEUROD1 (Fang et al., 2010). Together with our observation that NEUROD1 is the most enriched TF in N-d41 (Fig. 2B), our TF network analyses suggest an epigenomic mechanism for the reported NEUROD1/NEUROG2-induced rapid differentiation of excitatory neurons (Zhang et al., 2013).

## 4. Discussion

Studying temporal epigenetic regulation can help understand the molecular mechanisms of neurodevelopmental disorders such as autism (Nagode et al., 2017). Whilst the temporal dynamics of open chromatin states and TF networks have been studied for mouse neurodevelopment, and human stem cell differentiation into neural progenitor cells (Preissl et al., 2018; Ziller et al., 2015; Wilken et al., 2015; Podobinska et al., 2017), the chromatin accessibility dynamics and core TF networks, especially as assayed by ATAC-seq, for hiPSC neuronal differentiation has not previously reported. Here, using glutamatergic neurons differentiated from hiPSCs as a model, we carried out an integrated analysis of the dynamic changes of genome-wide OCRs (using ATAC-seq) and transcript abundances (using RNA-seq) during neuronal differentiation. We found that changes in OCR accessibility were positively correlated with changes in mRNA abundances, and the dynamic changes of OCR were accompanied by the binding of cell stage-specific TFs. Analyzing the cell stage-specific TF networks and their master regulators further supported the pivotal role of NEUROD1 and NEUROG2 in excitatory neuronal differentiation from hiPSCs. Altogether, our results show that the dynamics in OCR and TF networks contribute to regulation of key neurodevelopmental stages, which provides a mechanistic understanding of the epigenetic control of neuronal development from hiPSCs. Such knowledge may be instrumental for using hiPSC to generate cellular models of neuropsychiatric diseases.

While neurons differentiated from patient-specific hiPSCs are becoming a promising cellular model to de-convolute neurodevelopmental disorders, the epigenetic control of lineage-specific differentiation of neuronal cells at promoter accessibility level remains less known. As expected and consistent with previous findings on non-neuronal lineage cell differentiation (Wu et al., 2016; Pliner et al., 2017), we observed concordant dynamic changes of promoter/TSS chromatin accessibility of a gene and its expression during hiPSC neuronal differentiation. For the first time, we have interrogated the dynamic changes of genome-wide chromatin accessibility to TFs during neuronal differentiation from hiPSCs and have identified potentially important TFs implicated in neural lineage development but not previously associated with chromatin regulation of lineage determination. As expected, we noticed that genes characteristic of ES cells for maintaining pluripotency and self-renewal, such as NANOG and POU5F1 (OCT4), have their TFBFs most enriched at the hiPSC stage. Moreover, the upstream OCRs of these pluripotency genes, which are highly active and accessible at the hiPSC stage, switch to an inactive state at the N-d30 stage. On the other hand, also as expected, TFBFs that are known to be critical for glutamatergic neuronal differentiation are most enriched in neurons. For example, in the assembled key TF network of N-d41, gene targets of NEUROD1 and ASCL1 are extensively correlated to cell cycle and mitosis (Augustyn et al., 2014; Pattyn et al., 2004), while gene targets of LHX2 and VAX2 are more connected to neural functioning (Fig. 3B) (Zhang et al., 2014; Bulchand et al., 2001; Perez et al., 2012; Barbieri et al., 1999), reflecting their different functional aspects during neurodevelopment. Though interesting, these TFBS were nonetheless obtained from motif-based PIQ prediction, for which the validity of some observations remains to be empirically tested by ChIP-seq.

Notably, our ATAC-seq OCR analysis and the neuronal TF network analyses supported a known central role of NEUROD1 and NEUROG2 in glutamatergic neuronal differentiation from hiPSCs. Both TFs have been previously reported for their abilities in the rapid induction of stem cells into excitatory neurons (Zhang et al., 2013). However, the molecular mechanism underlying such processes remains vague. Here, we report that NEUROD1 binding footprints are most enriched at the N-d41 stage, forming a TF network with ASCL1, EMX2, and POU3F2 at the N-d41 stage (Fig. 3B). Similarly, NEUROG2 serves as a shared target of EMX2, ASCL1, and LHX2 within the same N-d41 TF network and as a target of INSM1 in the N-d30 TF network as well. It is noteworthy that the NEUROD1 sub-network seems to have multifaceted roles: (1) its targets are enriched in GO-terms of the cell cycle, transmembrane transport, and microtubule-based movement; (2) some other specific targets have been known to play important roles in neuronal differentiation and maturation. For instance, GPC3 encodes a presynaptic proteoglycan involved in synapse development, and FAT4 participates in neural migration (Zakaria et al., 2014; Song and Kim, 2013). Interestingly, we found that NEUROD1 targets itself at the promoter region (Fig. 4C), suggesting a possible self-regulation of NEUROD1 expression during neuronal differentiation. Similarly, several NEUROG2 target genes also participate in neuronal differentiation and maturation. For example, the role of ERBB4 in bipolar disorder and major depression has been widely reported (Chen et al., 2012; Goes et al., 2011). In addition, PARD3 and HDAC9 have been both implicated in mental disorders such as schizophrenia (Gao et al., 2018; Kim et al., 2012; Lang et al., 2012). Our study thus provides a mechanistic understanding at epigenetic level (chromatin accessibility) and from TF network perspective on the known function of NEUROD1 and NEUROG2 in rapid excitatory neuron differentiation.

We acknowledge that our study has some limitations. First of all, our analyses were based on open chromatin dynamic changes of a single hiPSC line (although with replicates at each assayed point), which did not account for possible inter-individual variations of OCRs. Secondly, the OCR dynamics may vary between different differentiation methods. However, we have previously shown that the global OCR profiles of neurons (N-d30) derived from this specific hiPSC line are very similar to that of NGN2-induced excitatory neurons (day 15) from a different individual (Forrest et al., 2017). Thirdly, although very similar to embryonic stem cells (ESCs), hiPSCs have been shown to possess some epigenetic features acquired during the reprogramming process or as remnants of epigenetic memory of the donor tissue. As a result, some of the observations here may not apply to ESCs. Future studies to compare the temporal differences of OCR dynamics and TF networks between ESC and hiPSCs may help with our mechanistic understanding of the neuronal differentiation and to improve the neuronal differentiation protocols. Furthermore, our assayed time points of neuronal differentiation are not exhaustive, e.g., N-d41 neurons are still relatively immature, assaying additional time points may provide a more comprehensive view of the OCR dynamics of neuronal differentiation. Finally, the neuronal differentiation protocol used in the present study does not involve TF overexpression. An interesting follow up would be to understand how TF may influence chromatin remodeling by knocking down or overexpression cell stage-specific TFs identified in this study. Therefore, future biological validation of the open chromatin dynamics and the implicated TF networks during neurodevelopment in additional samples and ESCs, under different differentiation conditions with more temporal assay points will further consolidate their roles during neuronal differentiation. It would also be interesting to replicate our study in a differentiation protocol targeting different neural cells, e.g., the GABAergic interneurons via exogenous expression of ASCL1 and DLX2 (Yang et al., 2017), to determine if similar mechanisms are involved.

Amid limitations, our global open chromatin profiling provides insight into the epigenomic control of hiPSC-derived glutamatergic neuronal differentiation at the level of chromatin accessibility. In addition, our results suggest that the modification of the main OCRs or key nodes within the TF network may alter the course of neural development. Our results and together with future biological validation of the importance of such OCRs or TF network nodes (e.g. TEAD1 in N-d30 TF network) in neurodevelopment, may help optimize the hiPSC-neuron disease models for understanding the molecular basis of neuropsychiatric disorders at the level of transcriptional regulation and chromatin remodeling.

## Supplementary Material

1

2

3

4

5

6

7
