# Supplementary material for: Open chromatin dynamics reveals stage-specific transcriptional networks in hiPSC-based neurodevelopmental model
Source: Stem Cell Res. Author manuscript; Available in PMC 2018 Jun 29. (PMC6025752; doi:10.1016/j.scr.2018.03.014)
Supplement: 1 [file NIHMS975267-supplement-1.docx]

# **Supplementary Materials**

# Supplementary Figure Legends

**Figure S1: IF staining of N-d41 glutamatergic neurons.** (A) MAP2 (green), PSD-95 (red), DAPI (blue); (B-D) VGLUT1 (green), DAPI (blue).

**Figure S2: GO-term enrichment analysis of dynamically changing open chromatin peaks during cortical neuronal differentiation.** Up=Increased openness, down=decreased openness, flat=unchanged. The openness change was quantitatively determined by using EdgeR to compare the difference of normalised reads number of the hotspot-called open chromatin peaks between iPSC, iN-d30 and iN-d41. The number of peaks showing dynamic change from iPSC to neurons was listed in the table and used for GO-term (biological process) enrichment analysis by DAVID. The enriched GO-terms (FDR < 0.05) were clustered and visualized by REVIGO. “Down-down” peaks were not used for DAVID analysis due to few number of peaks.

**Figure S3: Pearson correlation of expression folds (RNA-Seq) and peak counts at promoters (ATAC-Seq).** The Pearson’s correlation between different samples are shown in the upper panel with corresponding values described as a pie chart in the lower panel. Red: negative correlation; Blue: positive correlation.

**Figure S4: Expression changes of stage-specific TFs during iPSC differentiation.** (A) iPSC specific TFs with log_2_RPKM> 0.1 (*n* = 2); (B) N-d30 -specific TFs with log_2_RPKM> 0.1 (*n* = 3); (C) N-d41 specific with log_2_RPKM> 0.1 (*n* = 7).

# Extended Experimental Procedures

**1. iPSC lines and cell culture**

The iPSC line used in open chromatin mapping was derived from fibroblasts of a female subject GM01835 as previously described [1]. The study has been approved by the NorthShore University HealthSystem Institutional Review Board (IRB).

iPSCs were cultured using feeder-free method on Geltrex (Thermofisher)-coated plate in mTeSR1 media (StemCell). Media were changed daily, and cells were passaged as single cell every 4-6 days using Accutase (Thermofisher) in mTeSR1 in the presence of 5 µM ROCK inhibitor (R&D Systems).

**2. Cortical neuron differentiation from iPSC**

We have followed the protocol [2, 3] with minor modifications to fit our iPSCs growing in mTeSR. In brief, 5 wells of iPSCs (from a 6-well plate) were plated into one well of Geltrex (Thermofisher)-coated 12-well plate in mTeSR media. Cells were allowed to reattach overnight and reached 100% confluence 1 day post plating. For neural induction, cell culture media was switched to neural induction media (NIM) containing 1 µM of AMPK inhibitor Dorsomorphin and 10 µM of ALK inhibitor SB431542. A homogeneous neuroepithelial layer was formed 10 days post neural induction. Cells were then passed into laminin-coated 35-mm dish with culture media switched to Neural Maintenance Medium (NMM). Upon appearance of neural rosettes, 20 ng/ml of FGF2 were added for 2-4 days to promote the expansion of neural stem cells (NSCs). Approximately 20 days post neural induction, the first NSC expansion (1: 3) was performed and the second round of NSC expansion was performed at day 25. At day-30, the NSC culture was re-plated using Accutase at 50,000 cells per cm^2^ into laminin-coated 35-mm dish (for ATAC-seq) and laminin-coated coverslips (for immunofluorescence staining). We continued the neural differentiation for the plated cells in NMM until the specified days to harvest the cells for ATAC-seq or RNA-seq.

**3. DNA extraction, library preparation and sequencing of ATAC-seq**

For ATAC-seq, we harvested ~50,000 cells (iPSCs, induced NSCs and neurons at days 27, 33 and 41 of neural induction) by centrifugation at 500 g x 5 min at 4°C. Cells were washed once with PBS and pellet resuspended in 50 μL of cold lysis buffer (10 mM Tris-HCl, pH7.4, 10 mM NaCl, 3 mM MgCl_2_, 0.1% IGEPAL CA-630) to obtain cell nuclei. Nuclei were further isolated by spinning at 4°C with the supernatant discarded and immediately subjected to transposition reaction. We used a 50 μl transposition reaction system incubated at 37°C for 30 min, followed by immediate purification using a Qiagen MinElute Kit. Purified DNA was stored at -20°C until sequencing library assembly.

The library was generated and sequenced at University of Minnesota Genomic Center using a previously described protocol [4] except for (1) standard Nextera primer set were used for PCR amplification of the transposed DNA fragments (2) the optimum number of additional cycles for full PCR amplification was identified using qPCR, and (3) Agilent High Sensitivity DNA chip was used for library validation. All 6 libraries (2 for iPSC, 1 for N-d27, 1 for N-d33, and 2 for N-d41) were pooled and sequenced on a HiSeq 2500 using a paired-end (PE) 2x50 bp flow cell in a single lane. We achieved >120 million passing-filter reads for the lane, and the average quality scores for all libraries are all above Q30 for both R1 and R2 reads.

**4. ATAC-seq reads processing and alignment**

The paired-end reads were aligned to the human 1000g genome with Bowtie2 [5] with default parameters. Duplicated reads were removed using Picard function “MarkDuplicates”. Only paired-end reads that uniquely mapped were used for analysis. The combined aligned reads were used for hotspot and Protein Interaction Quantification (PIQ) analysis.

**5. ATAC-seq peak-calling**

We used Hotspot [6] to call open chromatin peaks for each ATAC-seq sample (2 for iPSC, 1 for N-d27, 1 for N-d33, and 2 for N-d41). Hotspot analysis was run for each sample to identify regions of enriched sequence tags. For the definition of hotspot (peak) size, we applied a minimum window size of 100 bp and maximum window size of 300 bp. Hotspot calculates the enrichment of sequence tags along the genome in the defined small window relative to a local background model based on the binomial distribution and using the observed tags in a 50-kb surrounding window as a background, and a false discovery rate (FDR) of 5% was used as a cut-off in peak calling [4]. The resulting hotspots were merged if more than 75 bp of overlap was present. To obtain a unique list of peaks, called hotspots in each sample were intersected between replicates, and only overlapping hotspots present in both replicates were kept, generating a final output of unique peaks that can be compared between different samples and cell stages.

**6. Analysis of cell type-specific peaks by GREAT and enriched TFBSs within by DAVID.**

For GREAT (version 3.0) analysis, N-d41-specific peaks (peaks that were found in the N-d41 group but not in either the N-d30 or iPS group) were isolated and assembled as .BED file as GREAT input. The minimum BED file contains the genome coordination of each N-d41 peak (FDR < 0.1) in one line. A total of 5,006 peaks were extracted for analysis. Parameters used: Human GRCh37 genome; no background regions as input; genomic regions association used “single gene mode” within 2,500 kb upstream/downstream. For DAVID (version 6.7) analysis, the same 5,026 N-d41 unique peaks were used. Each peak was associated to their nearest gene within 2500 kb upstream/downstream region. The gene names are assembled and served as the input for DAVID (<https://david-d.ncifcrf.gov/summary.jsp>) to generate p value and Fold Enrichment (FE) level.

**7. Quantification of chromatin accessibility of core promoters using EdgeR**

HOMER was used to annotate the RefSeq transcripts (hg19/GRh37). 8,626 open chromatin peaks that overlap with core promoter (annotated as Promoter-TSS) were identified. EdgeR [7] were used to quantify the differences in chromatin accessibility between iPSC and N-d30, taking the number of reads under each peak as inputs. An FDR of 5% was used as a cut-off to determine the significance of the differences in chromatin accessibility. We identified 1,071 core promoter peaks became less accessible in N-d30 whilst 234 became more accessible (FDR < 5%).

**8. TF motif enrichment analysis by HOMER**

HOMER [8] was used to estimate the enrichment of TF motifs within cell stage-specific open chromatin peaks. A total of 264 HOMER-curated TF motifs were used for analysis. Intervals of different sets of open chromatin peaks were used as input: iPSC=8,861; N-d30=26,012; N-d41=5,006; N-specific=43,916; and iPSC/N shared=18824. For each set of peaks and the corresponding given motif sequence, HOMER gave p-value, the percentage of peaks (target sequences) with a specific motif and background sequences (target sequences) with a specific motif, as well as an associated p-value for motif enrichment. We adjusted p-value by Bonferroni correction and calculated the fold of motif enrichment by dividing the percentage of peaks (target sequences) with a specific motif by the background sequences (target sequences) against a specific motif. To visualise relative motif enrichments, the fold of TF motif enrichments of each cell stage were plotted heatmap for TFs with a significance of enrichment at *P* value < 10^-20^ (Bonferroni corrected) in iPSC, N-d30 and N-d41specific peaks as well as the shared peaks by N-d30 and N-d40 (N) or by iPSC and Ns (iPSC/N).

**9. TF-binding footprint enrichment analysis by PIQ**

We compiled a set of motif in JASPAR formatted motif file to include more motifs from HOMER [8], which has 1,316 TF motifs initially used by PIQ [9] and 45 non-overlapping ChIP-Seq TF motifs from HOMER that were highly enriched in cell type-specific open chromatin peaks. BAM files were used as input, and we combined reads of 2 replicates for each cell stage (iPSC, N-d30 and N-d41). PIQ outputs a set of binding site calls and purity scores (corresponding to FDR).

To analyse the cell type/stage-specific TFs, we used a purity score of 0.9 as a cut-off (FDR=0.1). 1356/1361 analysed TFs had >1 footprint in at least one cell type, resulting in 2.1, 2.9, and 2.2, million TF footprints for iPSC, N-d30, N-d41, respectively. To calculate the ratio of TF footprints between different cell types, a constant number of 1 was added to the counts of TF footprints. We calculated the relative enrichment p-values between cell lines by Fisher’s exact test and Bonferroni corrected by the number of the tested TFs. A total of 300 TFs were identified specific to iPSC (n=7), N-d30 (n=33), N-d41 (n=185) or shared by N-d30/d41 (n=74) as defined by using a cut-off of more than 2-fold difference of the number of TF footprints between cell types (Bonferroni corrected *P* < 0.05; Fisher exact test). For determining TFs shared by N-d30/N-d41, the ratios of TF footprints in N-d41/N-d30 were normalised the counts of a TF in each cell stage by the total number of TF binding footprints. HOMER was used to create density plots of sequence tags around a motif region. Aligned reads were processed with the makeTagDirectory command. The annotatepeaks.pl script was used to generate the plots with the parameters –size 1000 –hist 1.

**10. Immunocytochemistry**

For routine characterisation of NSCs, cells were fixed in 4% PFA (Sigma) for 15 min at room temperature. After 3 times brief wash in PBS, cells were permeabilised with 1% Triton X-100 (Sigma) in PBS for 15 min at room temperature and blocked in 3% BSA and 0.1% Triton X-100 in PBS at 4°C overnight. Then the samples were incubated with primary antibodies at 4°C overnight, followed by 3 times of PBS wash. The samples were then incubated with secondary antibodies at room temperature for 1hr. After another 3 times PBS wash, samples were incubated in 2.5 μg/ml DAPI (4', 6-diamidino-2-phenylindole) at room temperature for 10min and mounted on glass slides. We prepared antibodies in blocking solution. The images were taken using a Nikon ECLIPSE TE2000-U microscope. Primary antibodies used and their dilutions for incubation were: Otx 1/2 (Millipore, 1:300) and Nestin (Abcam, 1:300) Secondary antibodies were 1:800 diluted (Alexa 488, Jackson ImmunoResearch).

For immunostaining of N-d30 neurons for GFP, PSD-95 and vGLUT1 for visualisation of dendritic and synaptic analyses, cells were fixed using 4% PFA (Sigma) in 4% sucrose/PBS for 15 min at room temperature. Cells were permeabilised and blocked simultaneously in PBS containing 0.1% Triton and 5% normal goat serum (Jackson Immunoresearch) for 2 h at 4°C followed by incubation with primary antibodies overnight at 4°C. Cells were washed three times in PBS and incubated with secondary antibodies at room temperature for 1hr followed by another three washes in PBS. Coverslips with immunostained cells were briefly washed in distilled water and mounted onto microscope slides using Prolong anti-fade reagent (Life Technologies). Primary and secondary antibodies were diluted in PBS containing 5% normal goat serum. Neurons from the same differentiation experiment were fixed and stained at the same time with identical antibody dilutions. Primary antibodies: GFP (Abcam, 1: 10000), vGLUT1 (Synaptic Systems, 1: 10000), and PSD-95 (NeuroMab 1: 1000). Secondary antibodies: Alexa 488 donkey anti-chicken (1: 1000), Alexa 568 donkey anti-mouse (1:1000), Alexa 647 donkey anti-rabbit (1: 1000).

**11. RNA sequencing (RNA-seq) and data processing**

We carried out RNA-seq for iPSC, N-d30 and N-d41 during cortical neuron differentiation. Total RNAs were isolated by using MirVana kit (Thermofisher) with RNA integrity number (RIN) were all > 8. The sequencing library was prepared with TruSeq stranded RNA library preparation reagents. RNA-seq was carried out at the University of Minnesota Genomics Center (UMGC) on an Illumina HiSeq 2500 using v4 chemistry. The average quality score is above the Q score cut off of 30 in all libraries. The total number of reads were 31-35 million of single 50-bp reads/sample for the cells used for ATAC-seq (2 iPSC, 2 N-d30, and 1 N-d41). We aligned the 50-bp single reads to the human reference gene map (GENCODEv18) using the Tophat v2.0.5 [10], allowing for 2 mismatches. We counted the raw reads by using the HTseq-count script ([www-huber.embl.de/users/anders/HTSeq/doc/overview.html](http://www-huber.embl.de/users/anders/HTSeq/doc/overview.html)) [11] and calculated gene level expression as RPKM [12] based on the exon model of the longest transcript of a gene (GENCODE v18). We then quantile-normalised RPKMs. We only analysed autosomal genes with log_2_(RPKM)>0.1 in iPSC and N-d30, which gave a list of 34,298 expressed genes. We used EdgeR to examine the differential expression between N-d30 and iPSC (each with two replicates), and we corrected for multiple testing by FDR.

**12. Counting shared genes and shared enriched GO terms.**

In order to generate the list of shared genes between ATAC-Seq and RNA-Seq data at different stages, we firstly annotated the peak lists from different groups and added their associated gene names. For ATAC-Seq peaks inside an annotated gene, the gene name was used. For peaks located outside annotated genomic regions, the nearest gene was associated. For RNA-Seq results, we selected all genes with more than 2 raw counts and analysed their expression dynamics using EdgeR. To determine the dynamics of gene expression level, we used output from EdgeR by defining group name as follows: Up: increased expression level with statistically significance (EdgeR, p < 0.05); flat: no statistically significant changes in the expression level were observed; down: decreased expression level with statistically significance. The number of shared genes from each group was counted in R, and the shared gene lists were used as input for DAVID GO term analysis (biological processes). Out of the 5 groups of shared gene lists used, DAVID had only two groups returned with significantly enriched GO term list (Table S5 and S6).

**13. Assembly and visualisation of TF regulatory network using CytoScape**

TF networks at N-d30 and N-d41 were assembled firstly by extracting master nodes using data generated by PIQ to form the most connected TF network with a cut-off score of 0.9. Gene expression level were extracted from RNA-Seq data and assigned to each TF in the list correspondingly. Tab-delimited, multi-column data was used as input for CytoScape.

**Supplemental References**

[1] S. Shi, C. Leites, D. He, D. Schwartz, W. Moy, J. Shi, J. Duan, MicroRNA-9 and microRNA-326 regulate human dopamine D2 receptor expression and the microRNA-mediated expression regulation is altered by a genetic variant, J Biol Chem, 289 (2014) 13434-13444.

[2] Y. Shi, P. Kirwan, J. Smith, H.P. Robinson, F.J. Livesey, Human cerebral cortex development from pluripotent stem cells to functional excitatory synapses, Nat Neurosci, 15 (2012) 477-486, S471.

[3] Y. Shi, P. Kirwan, F.J. Livesey, Directed differentiation of human pluripotent stem cells to cerebral cortex neurons and neural networks, Nat Protoc, 7 (2012) 1836-1846.

[4] J.D. Buenrostro, P.G. Giresi, L.C. Zaba, H.Y. Chang, W.J. Greenleaf, Transposition of native chromatin for fast and sensitive epigenomic profiling of open chromatin, DNA-binding proteins and nucleosome position, Nat Methods, 10 (2013) 1213-1218.

[5] B. Langmead, C. Trapnell, M. Pop, S.L. Salzberg, Ultrafast and memory-efficient alignment of short DNA sequences to the human genome, Genome Biol, 10 (2009) R25.

[6] S. John, P.J. Sabo, R.E. Thurman, M.H. Sung, S.C. Biddie, T.A. Johnson, G.L. Hager, J.A. Stamatoyannopoulos, Chromatin accessibility pre-determines glucocorticoid receptor binding patterns, Nat Genet, 43 (2011) 264-268.

[7] M.D. Robinson, D.J. McCarthy, G.K. Smyth, edgeR: a Bioconductor package for differential expression analysis of digital gene expression data, Bioinformatics, 26 (2010) 139-140.

[8] S. Heinz, C. Benner, N. Spann, E. Bertolino, Y.C. Lin, P. Laslo, J.X. Cheng, C. Murre, H. Singh, C.K. Glass, Simple combinations of lineage-determining transcription factors prime cis-regulatory elements required for macrophage and B cell identities, Mol Cell, 38 (2010) 576-589.

[9] R.I. Sherwood, T. Hashimoto, C.W. O'Donnell, S. Lewis, A.A. Barkal, J.P. van Hoff, V. Karun, T. Jaakkola, D.K. Gifford, Discovery of directional and nondirectional pioneer transcription factors by modeling DNase profile magnitude and shape, Nat Biotechnol, 32 (2014) 171-178.

[10] C. Trapnell, A. Roberts, L. Goff, G. Pertea, D. Kim, D.R. Kelley, H. Pimentel, S.L. Salzberg, J.L. Rinn, L. Pachter, Differential gene and transcript expression analysis of RNA-seq experiments with TopHat and Cufflinks, Nat Protoc, 7 (2012) 562-578.

[11] S. Anders, W. Huber, Differential expression analysis for sequence count data, Genome Biol, 11 (2010) R106.

[12] A. Mortazavi, B.A. Williams, K. McCue, L. Schaeffer, B. Wold, Mapping and quantifying mammalian transcriptomes by RNA-Seq, Nat Methods, 5 (2008) 621-628.
