# Supplementary material for: Open chromatin dynamics reveals stage-specific transcriptional networks in hiPSC-based neurodevelopmental model
Source: Stem Cell Res. Author manuscript; Available in PMC 2018 Jun 29. (PMC6025752; doi:10.1016/j.scr.2018.03.014)
Supplement: 2 [file NIHMS975267-supplement-2.docx]

**Table S1:** List of GO term enrichments in gene groups with different patterns of dynamic changes of ATAC-seq peak.

| Group description (change of chromatin in N-d30 vs. iPSC then in N-d40 vs. N-d30) | GO term enriched (FDR < 0.05) |
| --- | --- |
| Down-flat | Metal ion transport;  Positive regulation of nitrogen compound metabolism;  Cell adhesion;  Biological adhesion;  Cell projection organization;  Cell fate commitment;  Embryonic morphogenesis;  Negative regulation of cellular biosynthesis;  Neuron differentiation;  Cell-cell signalling. |
| Flat-down | Regulation of growth;  Cellular component movement;  Positive regulation of macromolecule metabolism;  Regulation of cellular component movement. |
| Up-flat | Positive regulation of gene expression;  Positive regulation of cellular biosynthesis;  Cell fate commitment;  Cell adhesion;  Forebrain development;  Biological adhesion;  Regulation of neurogenesis;  Neuron projection morphogenesis;  Cellular component movement;  Axonogenesis;  Cell morphogenesis;  Cell projection organization. |
| Flat-up | Cell morphogenesis involved in differentiation;  Biological adhesion;  Cell part morphogenesis;  Cell morphogenesis involved in neuron differentiation;  Neuron projection morphogenesis;  Cell adhesion;  Axonogenesis;  Neuron differentiation;  Regulation of neuron differentiation;  Synaptic transmission;  Cell-cell signalling. |
| Up-up | Biological adhesion;  Cell projection organization;  Homophilic cell adhesion via plasma membrane adhesion molecules;  Single organismal cell-cell adhesion;  Axonogenesis;  Cell morphogenesis involved in neuron differentiation;  Neuron projection development;  Neuron differentiation;  Cell adhesion. |
